# Clinical case: successful birth as a result of the transfer of an embryo at the blastocyst stage from an oocyte obtained from a tumoral ovary using the OTO-IVM method

**DOI:** 10.3389/fmed.2026.1857432

**Published:** 2026-07-17

**Authors:** Yuliya Tatishcheva, Olga Lavrinovich, Dmitri Gerkulov, Alla Kalugina, Nataliya Slominskaya, Natalya Kuzminih, Oksana Pryadkina, Igor Berlev, Andrey Karitski

**Affiliations:** 1 Department of Obstetrics and Gynecology, N.N. Petrov National Medical Research Oncology Center, Saint Petersburg, Russia; 2 Reproductive Medicine Clinic, Skyfert Ltd., Saint Petersburg, Russia; 3 Department of Obstetrics- Gynecology and Neonatology, I.P. Pavlov First Saint Petersburg State Medical University, Saint Petersburg, Russia

**Keywords:** fertility preservation, live birth, OTO-IVM, ovarian cancer, time-lapse

## Abstract

**Background:**

Preservation of fertility for women of reproductive age with malignant ovarian tumors is a complex issue, as organ-preserving treatment may increase the risk of recurrence. Direct involvement of the ovary in the tumor process significantly limits the possibility of implementing the most effective methods: the stimulation of ovulation and cryopreservation of ovarian tissue due to the risk of contamination by tumor cells. The only possible method of preserving fertility in such cases may be the OTO-IVM (Ovarian tissue oocyte *in vitro* maturation) method. It is based on obtaining immature oocytes from tissue of ovary removed during surgical treatment and their subsequent maturation in vitro. Up to date, based on the literature, 6 children have been born using this method (in three cases, the female patients had an ovarian tumor). In all cases, embryos were transferred at the cleavage stage.

**Methods:**

We present the first case of a successful birth as a result of the implementation of the OTO-IVM fertility preservation program with embryo transfer performed at the blastocyst stage. A 29-year-old female patient with a recurrence of a borderline tumor in a single ovary underwent surgery to preserve the uterus and remove the tumor-affected ovary.

**Findings:**

22 cumulus-oocyte complexes were retrieved from the removed ovary. As a result of OTO-IVM, 12 metaphase II stage oocytes were obtained, which were fertilized using the intracytoplasmic sperm injection (ICSI) method; 3 blastocyst stage embryos were obtained and cryopreserved. The female patient was prescribed cyclic hormone replacement therapy. After 9 months, one embryo was thawed and transferred into the uterine cavity, resulting in clinical pregnancy. Normal delivery by way of natural maternal passages, a healthy boy was born at the term of 37 weeks and 6 days.

**Discussion:**

Our work confirms that the OTO-IVM method has high value for preserving fertility in female patients with malignant ovarian tumor. We have shown for the first time that prolonged cultivation of embryos to the blastocyst stage in OTO-IVM programs can lead to live birth. Cultivation to the blastocyst stage makes it possible to conduct preimplantation testing of embryos, which is extremely important for patients with genetically determined forms of cancer. It is necessary to continue research to improve the method and determine the indications and contraindications for its implementation.

## Introduction

The detection of malignant and borderline ovarian tumors among women of reproductive age is approximately 15% and represents one of the most difficult issues of fertility preservation due to the direct injury of the ovary by tumor, resulting in reduction of the follicular reserve (1).

Different histological and molecular subtypes of ovarian cancer have different prognoses for the course of the disease, and require different degrees of aggressiveness for treatment, which in turn determines the extent of surgical management as well as the need for chemotherapy.

Discussion of fertility preservation in women of reproductive age with suspected malignant tumors of ovaries begins with an assessment of the feasibility of organ-preserving surgical treatment, which is feasible in the early stages of epithelial cancer, as well as in cases of borderline and germ cell tumors of ovary. Due to the significant diversity of histological types and clinical manifestations of ovarian cancer, the decision to perform organ-preserving surgery is made individually, only if the female patient is interested in preserving fertility (2, 3).

However, fertility-preserving surgical treatment is associated with an increased risk of disease recurrence (4). The recurrence rate can exceed 50%, especially when performing ultra-organ-preserving surgery. In 87% of cases, relapse is localized in the area of the preserved ovary (5). It is important to emphasize that the fulfillment of the extent of fertility-preserving surgery did not have a significant impact on overall survival indicators (Relative Risk: 1.09, 95%; Confidence Interval: 0.66–1.77, *p* = 0.73) in multivariate analysis, whereas tumor stage (extent of tumor spread) and tumor grade are two independent factors that correlate with overall survival indicators (6). At the same time, performing fertility-preserving surgery does not guarantee the preservation of the follicular reserve due to coagulative damage to the ovary.

The “gold standard” of preserving fertility in women of reproductive age is cryopreservation of oocytes and/or embryos. Preference is given to superovulation stimulation in order to obtain mature oocytes (at the metaphase II stage) and their cryopreservation using the vitrification method or, if a partner is available, fertilization and obtaining embryos (7, 8). The IVM (*in vitro* maturation) method is used less frequently and is an alternative to standard stimulation. Its essence lies in the collection of immature oocytes in any phase of the menstrual cycle, followed by their maturation *in vitro* (9). The described ART (assisted reproductive technology) methods involve transvaginal puncture of follicles and cannot be implemented until the clinical situation is clarified (8). Passing a puncture needle through a tumor carries the risk of dissemination of cancer cells along the needle, which can cause implantation metastases and, as a consequence, disease progression (10). If postoperative histological examination confirms the malignant nature of the tumor, any manipulations that led to a violation of its capsule or integrity before surgery or intraoperatively are regarded as iatrogenic dissemination. This changes the stage of the disease toward progression and requires a revision of treatment tactics toward its intensification. If there are signs of malignant transformation of the tumor in the ovary, transvaginal puncture is contraindicated, just as cryopreservation of ovarian tissue does not seem possible.

A number of scientific studies examine the possibility of cryopreservation of oocytes or embryos obtained through the stimulation of ovulation in female patients with ovarian tumors. Most authors indicate that this procedure is permissible only after completion of the surgical stage of treatment under certain morphological characteristics of the tumor. This requires having the results of the final histological conclusion, data from a complete surgical staging of the disease, as well as a time reserve of at least 3–4 weeks (11–13). At the same time, the use of gonadotropic preparations in patients with hormone-sensitive ovarian tumors (borderline, low-grade epithelial, and germ cell) remains limited. This is due to their differentiated receptor status and uncertain risks of tumor growth during the stimulation of ovulation.

Thus, one of the safe and acceptable strategies for preserving fertility in this category of patients is the OTO-IVM (*Ovarian Tissue Oocyte In Vitro Maturation*) method, which is based on the IVM technique applied to immature oocytes obtained from ovarian tissue removed during surgical treatment (14). The method is simultaneous with the main treatment. In case of large tumor sizes and bilateral ovarian involvement, it appears to be the only possible method that will allow cryopreservation of biological material for use in ART programs.

By now, evidence of 6 successful live births in the world after OTO—IVM has been published in the literature. In female patients with Hodgkin’s lymphoma, breast cancer and Uterine Arteriovenous Mailformation, OTO-IVM programs were performed under cryopreservation of cortex of intact ovaries. Effective oocyte maturation was 32, 38, and 46%, respectively. In all cases, the embryos were cryopreserved and then thawed and transferred into the uterine cavity at the cleavage stage, resulting in the birth of two girls and a boy (15).

The remaining 3 cases of live birth after OTO-IVM were cases of birth of a child from an oocyte obtained from the ovary affected by the tumor. The first successful pregnancy and birth after OTO—IVM was in a 21-year-old female patient with a malignant tumor of the ovary (high-grade micropapillary serous ovarian carcinoma). Organ-preserving treatment was performed in the setting of right-sided salpingo-oophorectomy and cystectomy of the left ovary, followed by adjuvant chemotherapy. Seven months after completion of treatment, a relapse of the disease was diagnosed. A left-sided salpingo-oophorectomy with preservation of the uterus and OTO-IVM procedure were performed.

Subsequent embryo transfer resulted in a singleton pregnancy, which resulted in the birth of a healthy boy (16). The second birth occurred in a female patient who, at the age of 23, was diagnosed with a recurrence of borderline mucinous cystadenoma IA measuring 8 cm in a single left ovary. Surgical treatment was performed in the context of left-sided salpingo-oophorectomy, after which OTO-IVM was performed. Three embryos were obtained, which were cryopreserved at the zygote stage by slow freezing. After completion of treatment, 3 zygotes were thawed, cultivated to the cleavage stage, and two embryos (B8 and B7) were transferred. A healthy boy was born (17). The third birth after the OTO-IVM program is described in a female patient who, at the age of 33, experienced a relapse of a serous borderline tumor of the only right ovary and underwent a right salpingo-oophorectomy. OTO-IVM was performed; three embryos were cryopreserved by vitrification on day 3 (8-cell Veeck2, 4-cell Veeck2, 5-cell Veeck2). Three months later, the transfer of 1 embryo was performed in a cycle with hormone replacement therapy. At 36 weeks of pregnancy, an emergency cesarean section was performed; the condition of the newborn was satisfactory; no pathology was detected (18).

Thus, in all the cases described above, embryo transfer was performed no later than at the cleavage stage. This transfer strategy, on the one hand, allows placing the embryo in the natural conditions of the mother’s body faster and avoiding long-term impact of cultivation in vitro; on the other hand, it does not provide the opportunity to ensure that the embryo is capable of reaching the blastocyst stage. In addition, only prolonged cultivation allows performing trophectoderm biopsy and genetic analysis for aneuploidies and/or monogenic diseases, including genetically determined forms of cancer. Previously, it was reported that three euploid blastocysts were obtained in a 30-year-old patient with HER2-positive luminal breast cancer who underwent fertility preservation using the OTO-IVM method. The authors described extended culturing (the blastocysts were biopsied on day 7 of development), and this is certainly a very valuable experience, even though the embryos were not transferred to the patient (19). Up to date, there have been no reports of live birth from embryos obtained from OTO-IVM oocytes and transferred into the uterine cavity at the blastocyst stage.

We present the fourth case of successful pregnancy and delivery of a healthy baby in a female patient with a tumor of a single ovary, when a blastocyst-stage embryo transfer was performed for the first time. The aggregated data on all live birth cases in OTO-IVM programs are presented in Table 1, which was originally created by Higuchi et al. (18) and has been supplemented with our case.

**TABLE 1:** List of reported cases of live birth from OTO-IVM.

Authors	Maternal age		History	Immature oocytes	MII oocytes after IVM	Maturation rate (%)	Cryopreservation	2PN oocytes after ICSI	Embryo stage	Gestational age	Delivery mode	Weight of baby (g)	Sex of baby
	At OTO-IVM	At delivery											
Prasath et al. (16)	21	NA	Lt ovary recurrent serous borderline tumor	4	4	100%	Embryo	3	Cleavage stage	NA	NA	2,580	M
Uzelac et al. (17)	23	26	Lt ovary recurrent mucinous borderline tumor	10	4	40%	Embryo	3	Cleavage stage	Term	NA	3,883	M
Segers et al. (15)	23	26	Hodgkin lymphoma	22	7	32%	Oocyte	4	Cleavage stage	39w4d	VD	3,150	M
	26	27	Uterine Arteriovenous Malformation	13	6	46%	Embryo	3	Cleavage stage	38w2d	CS	2,660	F
	36	42	Breast cancer	8	3	38%	Embryo	3	Cleavage stage	40w6d	CS	3,860	F
Higuchi et al. (18)	34	36	Rt ovary recurrent serous borderline tumor	8	6	75%	Embryo	3	Cleavage stage	36w6d	CS	2,906	M
Present case	29	31	Lt ovary recurrent serous borderline tumor	22	12	55%	Embryo	10	Blastocyst stage	37w6d	NA	3,300	M

The rows highlighted in color correspond to ovarian cancer cases. The case described in this manuscript is indicated in red font. COC: cumulus-oocyte, VD: vaginal delivery, CS: Cesarean section. Adapted and supplemented from Higuchi et al. (18).

### Clinical case

Female patient E., born in 1994. The patient has a 3-year history of primary combined infertility in her gynecological background. She was under observation by a gynecologist for hyperprolactinemia and PCOS. She was taking Dostinex (cabergoline) at a dose of 0.5 mg twice a week. Ovulation stimulation was planned. During a follow-up examination in July 2021, a cystic mass of the right ovary measuring 36 × 27 mm with a solid component was identified by pelvic ultrasound. On 18.09.2021, surgical treatment was performed via laparoscopy, removal of a lesion of the right ovary (High-Grade serous carcinoma). She applied to the National Medical Research Center of Oncology for further treatment. The material was reviewed histologically, and a serous borderline tumor of the ovary was diagnosed. Due to the patient’s young age and unrealized reproductive function before the final diagnosis was established, a decision was made to preserve fertility. On 29.10.2021, a laparoscopic right-sided adnexectomy, resection of the left ovary, and surgical staging were performed. Final histological examination as of 08.11.2021: serous borderline tumor of ovary. The patient denied any family history of known oncological diseases. Blood testing for hereditary genetic mutations BRCA1 and BRCA2 by PCR dated 05.10.2021 was negative.

**FIGURE 1:**
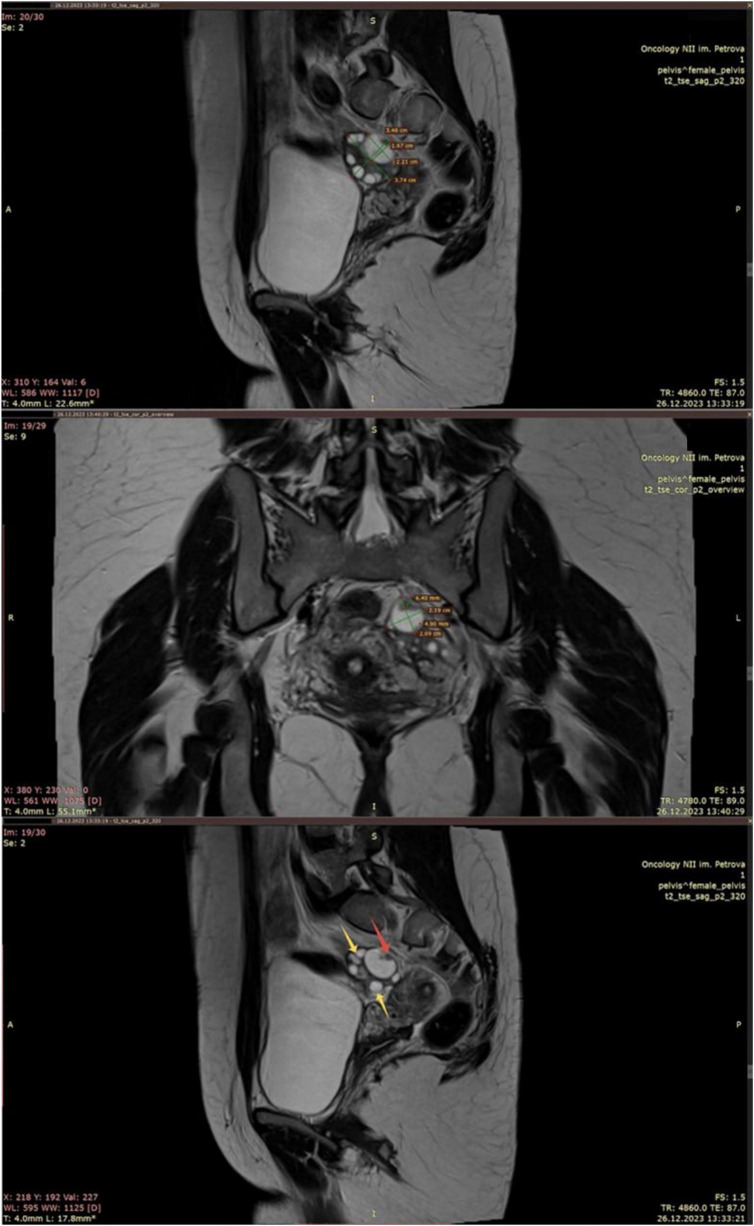
MRI image with intravenous contrast: recurrent tumor in the ovary with papillary growths along the internal capsule in two projections. MRI image with intravenous contrast: recurrent tumor in the ovary with papillary growths along the internal capsule in two projections. Red arrow shows recurrent tumor, yellow ones show antral follicles in the ovary.

In April 2023, a mass lesion was discovered in the left ovary. Case follow-up was performed according to the place of residence. In December 2023, the female patient sought examination at the National Medical Research Center of Oncology, where, based on the examination results, a recurrence of a borderline serous tumor (pT2bN0M0) in a single left ovary was diagnosed (Figure 1). At the tumor conference (17.01.24), a decision was made on surgical treatment in the scope of laparoscopic left-sided adnexectomy and preservation of the uterus, with the simultaneous implementation of the OTO-IVM method, which was performed on 21.02.2024. Histological examination (02.03.24) confirmed the presence of a recurrence of a borderline serous tumor in the left ovary against the background of a serous cystadenoma (ICD-O code 8442/6). The main events are presented in Table 2.

**TABLE 2:** The patient’s case history and timeline of events.

Date of surgery	Indication for surgery	Tumor markers	AMH	Clinic	Extent of surgical intervention	Results of histological examination	Disease stage (TNM)
18.09.21	Primary infertility, solid mass of the right ovary measuring 36 × 27 mm.	Were not detected	Were not detected	Republican Perinatal Center (Petrozavodsk)	Laparoscopy, removal of a mass from the right ovary, chromopertubation.	High-grade serous carcinoma	**c**T1aN0M0
October 2, 2021—referral to the N.N. Petrov National Medical Research Oncology Center, Ministry of Health of Russia (Saint Petersburg). Revision of the operative histological material on October 9, 2021: fragments of a serous borderline ovarian tumor.							
29.10.21	Fertility-preserving surgery, surgical staging	**CA**—125 = 25.79 U/mL	12.10.2021 = 7,17 ng/mL		Laparoscopic right adnexectomy with resection of the left ovary, omentectomy, multifocal peritoneal biopsies, and excision of the pouch of Douglas infiltrate	Borderline serous tumor of the ovaries with non-invasive implants in the pelvic peritoneum and pararectal peritoneum, measuring up to 5 mm in the greatest dimension.	Restaging**cT**2bN0M0
April 2023 (Petrozavodsk): A cystic mass in the left ovary was identified. Recurrence was suspected, and follow-up observation was recommended. September 22, 2023–Pelvic MRI with IV contrast: left ovary 5.4 × 2.4 × 3.7 cm with multiple follicles. One follicle, approximately 1 cm, shows parietal growths along the capsule with avid contrast uptake.							
December 26, 2023 (N.N. Petrov National Medical Research Oncology Center, Saint Petersburg)—Pelvic MRI with intravenous contrast: The left ovary measures 35 × 44 mm, with peripheral follicles up to 8 mm. Along the posterior wall, a cystic lesion measuring 23 × 15 mm is visualized, with an unevenly thickened capsule and a solitary parietal solid thickening measuring 7 × 5 mm, showing intense contrast accumulation. The right ovary has been removed.							
19.02.24	Recurrent tumor in the only remaining left ovary	**CA**-125–23,36 U/mL; AFP 2,95 IU/mL; b-ХГЧ < 0,1 IU/L; Inhibin B 62,1; pg/mL; LDH 161 IU/L.	12.01.2024 = 3,45 ng/mL	N.N. Petrov National Medical Research Oncology Center, Ministry of Health of Russia	Laparoscopy, left adnexectomy, OTO-IVM	Ovarian borderline serous tumor	Tumor recurrence **cT**2bN0M0

The patient with long-standing primary infertility was highly motivated to preserve her fertility. Organ-preserving surgery was a key condition for her adherence to treatment. Though aware of the limited efficacy and experimental nature of the OTO-IVM technique, the patient perceived undergoing fertility preservation via OTO-IVM as a significant psychological motivator for adhering to treatment and achieving satisfactory post-cancer rehabilitation.

### Retrieval and transport of ovary

**FIGURE 2:**
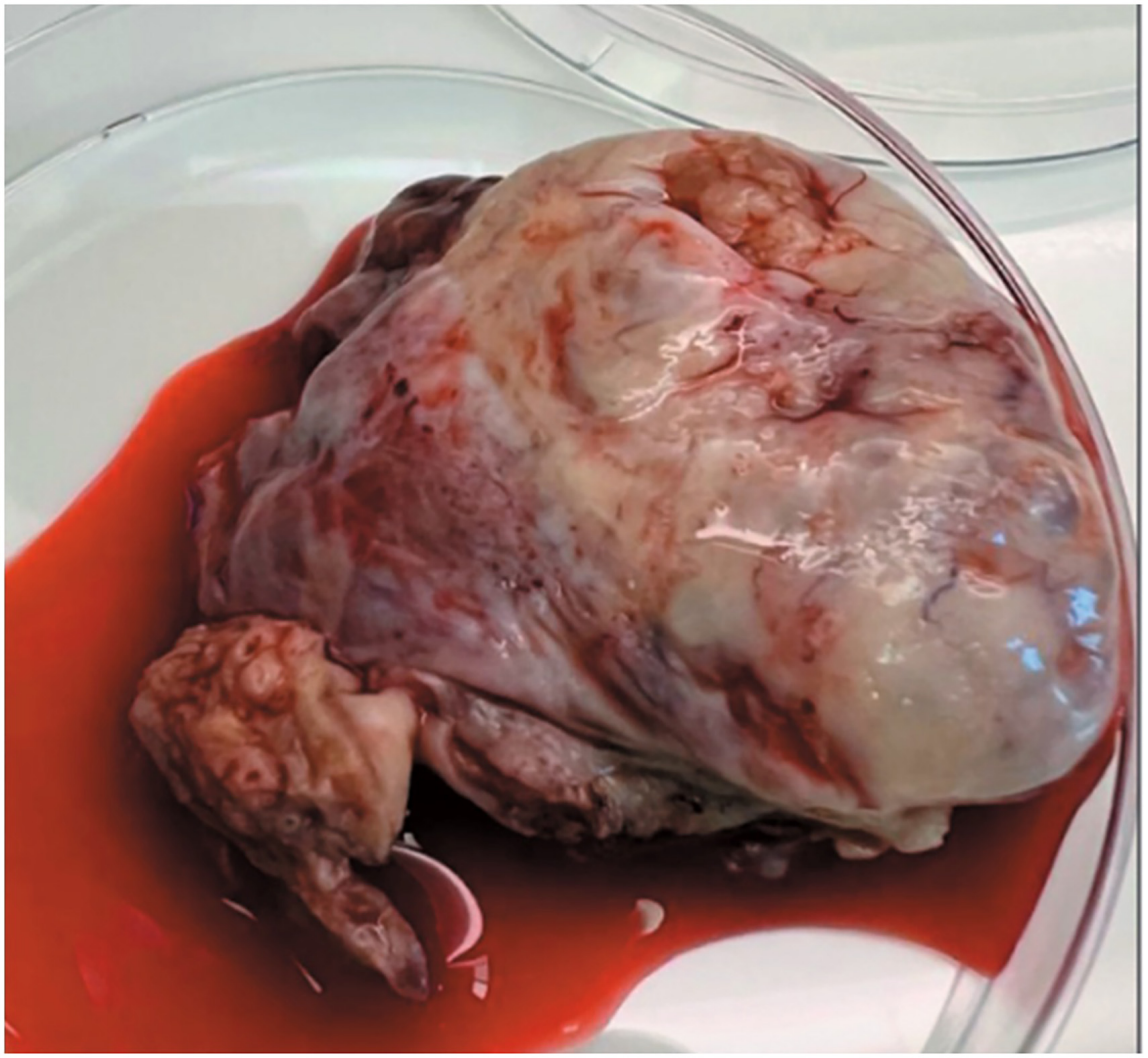
A single left ovary after adnexectomy.

Ovariectomy was performed at the National Medical Research Center of Oncology, and the ovary (Figure 2) was placed in a sterile container with Flushing Medium (Origio, CooperSurgical Inc., United States) heated to 37°C. The container was placed in a temperature-controlled transport box and was ferried to the laboratory within 70 min. Oocytes were obtained and further cultivated in the embryology laboratory of the Reproduction Clinic.

### Isolation of oocyte-cumulus complexes

After delivery to the clinic, all further manipulations with the tissue were carried out in a Flushing Medium (Origio, CooperSurgical Inc., United States) at + 37°C on a heated surface.

**FIGURE 3:**
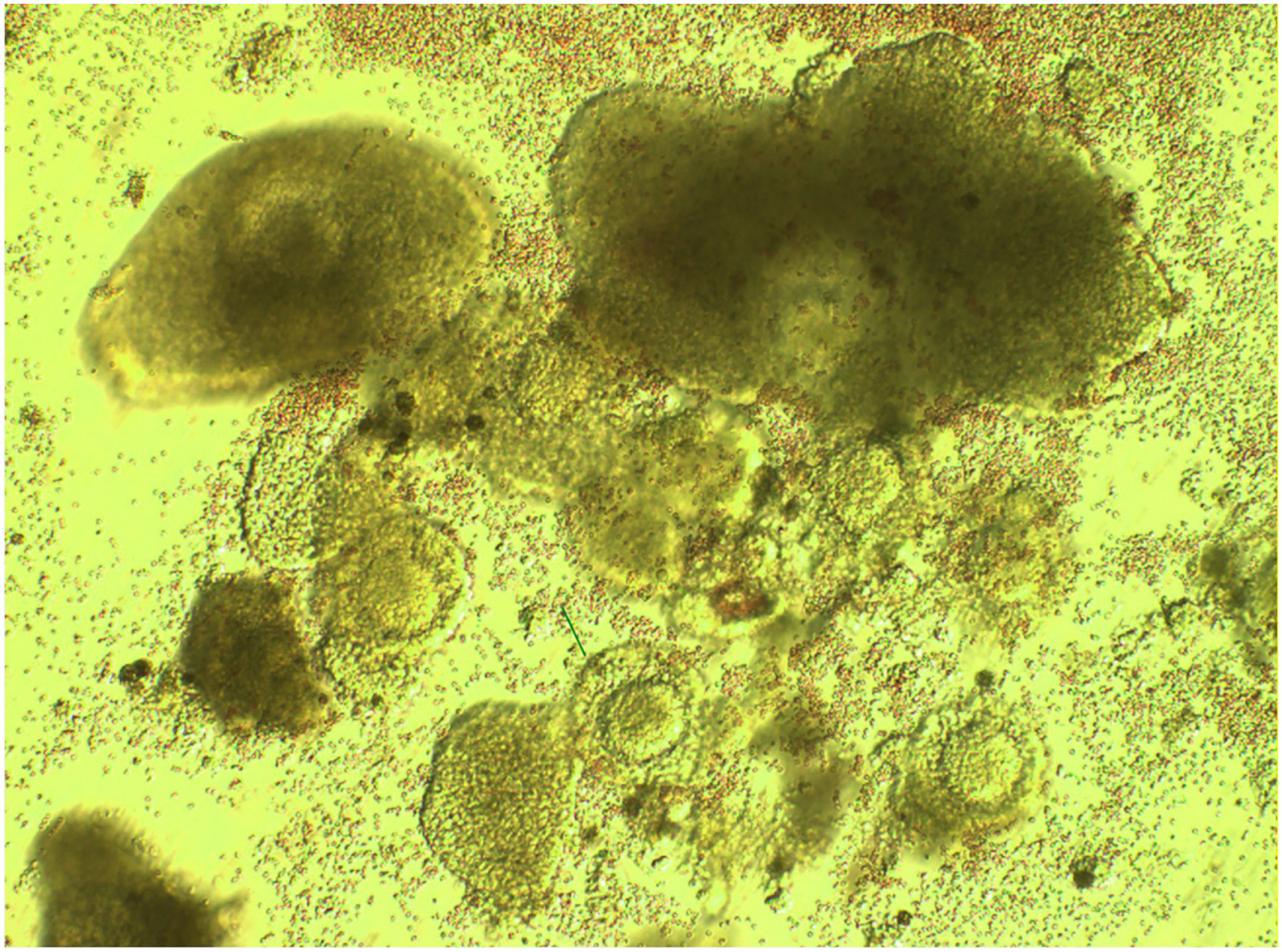
Oocyte-cumulus complexes after isolation. The image was taken under an Olympus IX73 inverted microscope (Olympus Corporate, Japan) at 200x magnification.

The ovary was transferred to a Petri dish and examined for the presence of antral follicles. When detected, all visible follicles were punctured with a 19G needle using a 2 ml syringe. The resulting follicular fluid was released into a Petri dish with Oocyte Washing Medium (Sage, CooperSurgical, United States) preheated to + 37°C and viewed under binocular magnifier (Olympus Corporate, Japan). The detected oocyte-cumulus complexes (OCCs) were transferred into a similar dish to wash off the follicular fluid (Figure 3). After aspiration of all visible follicles, the ovary was cut into two halves, each of which was transferred to a separate dish. Tumor tissue was identified in the ovarian medullary layer, whereupon it was excised and submitted for histopathological analysis. Next, longitudinal incisions were made with a sterile blade, both on the cortex side of the ovary and on the medullary side, to open the remaining follicles. The tissue was washed sequentially in several dishes with the medium, after which each dish was examined for the presence of OCC. Upon detection, OCCs were retrieved and transferred, like aspirated OCCs, into Oocyte Washing Medium (Sage, CooperSurgical, United States). The collected OCCs were evaluated under an Olympus IX73 inverted microscope (Olympus Corporate, Japan) at 200x magnification. Oocytes with the signs of atresia, with fragmented cytoplasm,

as well as empty pellucid zones without cytoplasm were considered degraded. Such oocytes were not used for further cultivation. Of the 22 OCCs obtained, 6 were degraded.

Upon completion, the ovarian tissue was placed in a sterile container with a 10% formalin solution and returned to the N.N. Petrov National Medical Research Center of Oncology for histological examination.

### Oocyte maturation

The remaining 16 OCCs were placed in a prepared in advance Oocyte Maturation Medium (SAGE, CooperSurgical Inc., United States) (8 h in a CO_2_ incubator at + 37°C) with the addition of 0.75 IU/ml FSH and 0.75 IU/ml LH in accordance with the manufacturer’s instructions under Mineral oil (Vitrolife, Sweden). Cultivation was performed in the Planer BT37 Mark II bench top multi-gas incubator (PLANER, CooperSurgical Inc., United States).

After 24 h, oocyte maturity was assessed at 200x magnification. Based on the presence of a polar body, 1 oocyte was detected at the metaphase 2 (M2) stage. The remaining OCCs continued to incubate, with an intermediate assessment performed after 36 h and a total incubation time of 48 h. After 36 h of cultivation, 9 more oocytes reached the M2 stage and 2 additional oocytes matured after 48 h. The detected mature oocytes were retrieved and cleaned of cumulus cells. The oocytes cleared of cumulus cells were assessed at 200x magnification, and the stage of oocyte development was recorded in the cultivation protocol: M2 (metaphase 2, mature oocyte)—12 oocytes, M1 (metaphase 1)—4 oocytes.

### Obtaining embryos

The ICSI fertilization procedure was performed twice: for 1 oocyte after 24 h of maturation and for 11 oocytes the next day (after 48 h of maturation). All 12 M2 stage oocytes were fertilized with the partner’s sperm using the ICSI method. Ejaculate parameters: concentration 33 million/ml, progressively motile 18%, total motility 33%.

**FIGURE 4:**
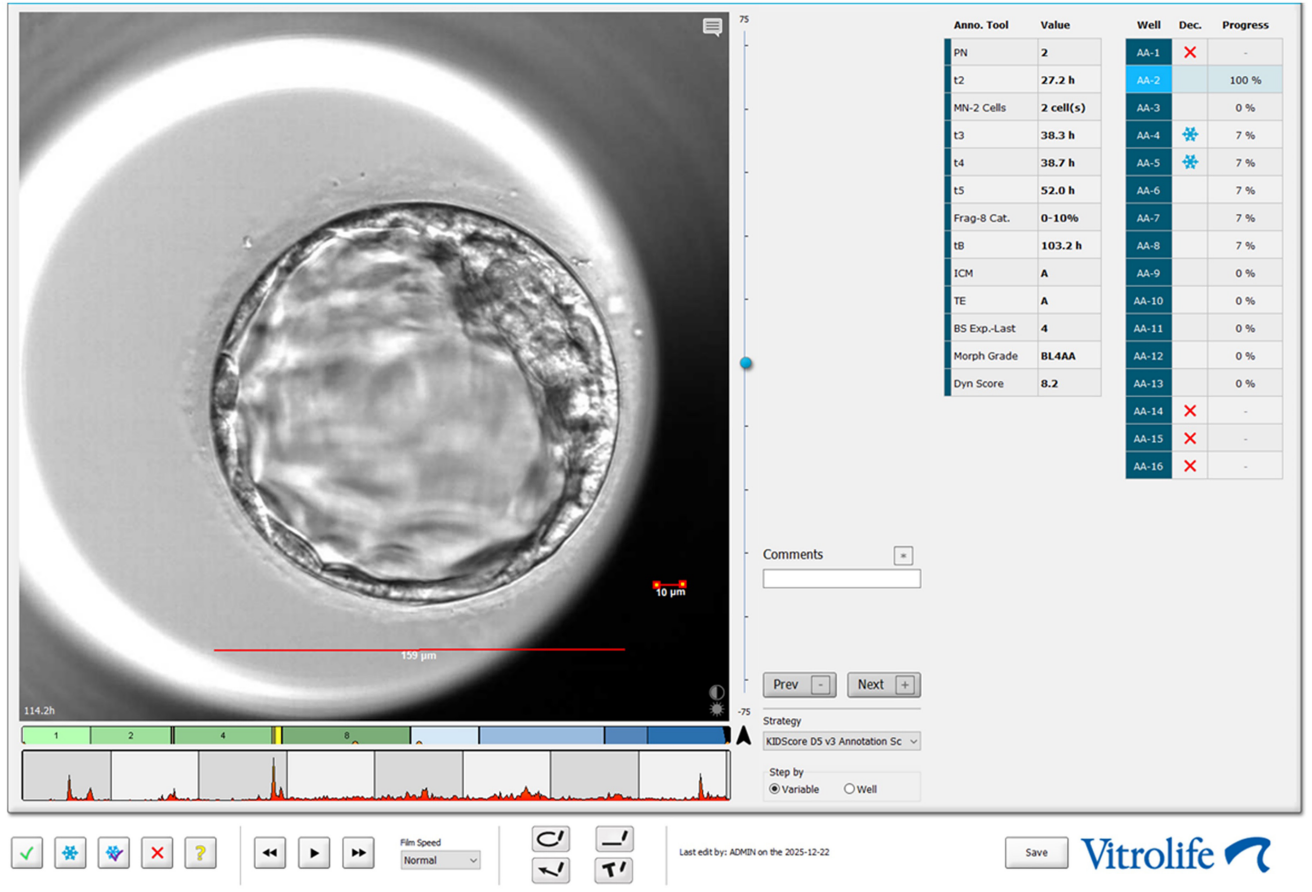
Embryo (4AA) at the blastocyst stage (day 5) before cryopreservation. The image from time-lapse incubator EmbryoScope Plus (Vitrolife).

After fertilization, oocytes were incubated in one-step GTL medium using Mineral oil (all are Vitrolife, Sweden). Ten zygotes were obtained and cultivated to the blastocyst stage. Embryo culture was conducted in a *time-lapse incubator EmbryoScopePlus* with the automatic annotation system *KIDScoreD5*. This algorithm takes into account the following morphokinetic parameters: the timing and uniformity of cleavage, the time of blastocyst formation, and the quality of the inner cell mass and trophectoderm. One embryo of excellent quality (4AA, *KIDScoreD5 is* 8.2 on a 10-point scale) (Figure 4) was obtained on the fifth day of cultivation from an oocyte that had matured after 24 h; two more embryos of good and satisfactory quality (2BB, 4BC) were obtained a day later from oocytes that had matured after 48 h.

The obtained embryos were cryopreserved by vitrification using a set of media for vitrification of oocytes and embryos (Kitazato Corporation, Japan).

### Fertility restoration program

Upon discharge from the inpatient facility, the female patient was prescribed combined hormone replacement therapy “Femoston 2/10” (2 mg Eestradiol and 10 mg Dydrogesterone, Abbott Biologicals B.V., Netherlands) in a continuous mode.

Nine months after the confirmation of disease remission using instrumental methods of diagnosis, a decision was made to attempt embryo transfer into the uterine cavity.

Thawing of the embryo (blastocyst 4 AA) was performed according to the standard method using appropriate media (Kitazato Corporation, Japan) 4 h before the planned transfer. Immediately upon thawing, the embryo was placed in one-step GTL medium using Mineral oil (all are Vitrolife, Sweden) and cultivated until the transfer to a Planer BT37 Mark II bench top incubator (PLANER, CooperSurgical Inc., United States). The embryo after thawing demonstrated complete survival, it was collapsed but had fully recovered by the time of transfer (Supplementary Figure 1).

The transfer of the thawed embryo was performed during a cycle of hormone replacement therapy. On the second day of the menstrual cycle, preparation of the endometrium with 6 mg/day of Estradiol valerate (Proginova, Zentiva Pharma LLC) was started. When the endometrial thickness reached 8 mm, micronized progesterone 600 mg per day, intravaginally, was added to estrogen therapy. The transfer of the thawed embryo was performed on the 5th day of progesterone administration. Hormone therapy was continued until ultrasound diagnosis of pregnancy, followed by gradual withdrawal.

Hormonal support was provided with Estradiol valerate preparations until the 14th week of pregnancy and micronized progesterone preparation, with a gradual reduction in dosage from the 24th to the 34th week of pregnancy.

The pregnancy proceeded without complications.

Uncomplicated vaginal delivery occurred at a term of 37 weeks and 6 days. A boy with an 8/9 according to the Apgar scale was born. At present, the child is healthy and developing physically and emotionally in accordance with age. The patient is under dynamic observation by an oncologist. No evidence of recurrence was found based on the results of the conducted examinations (pelvic and abdominal ultrasound, blood tests for tumor markers).

## Discussion

The OTO-IVM method is a promising way for preserving fertility when there is direct ovarian tumor involvement. It also appears valuable as a method for increasing the efficiency of cryopreservation of biological material during cryopreservation of ovarian tissue. Successful maturation and fertilization of oocytes in this case makes it possible to avoid invasive autotransplantation of cortex tissue.

However, OTO-IVM protocols for fertility preservation have not yet been standardized and differ in terms of the use or non-use of priming (the use of FSH and HCG preparations before puncture), the method of follicle puncture, the conditions for transporting tissue from the operating theater to the laboratory, and other parameters (20). While there are published cases of ovarian stimulation in the setting of ovarian cancers with subsequent retrieval of oocytes either via laparotomy or immediately after oophorectomy, in this case the oncology team did not feel such an approach would be safe for this patient.

It should be noted that one of the decisive aspects of successful preservation of oocytes for their maturation, subsequent successful fertilization, and embryo development is the condition of transportation of the ovary from the operating theater to the laboratory where the oocytes will be retrieved. Numerous studies confirm the damaging effects of low temperatures on the oocyte cleavage spindle. It has been shown that cooling to 0 C for 10 min completely and irreversibly destroys the oocyte cleavage spindle (21, 22). Therefore, we performed all procedures, starting from the moment the tissue was collected in the operating theater, at a constant temperature of 37°C. In addition, work with the ovarian tissue in the embryology laboratory began within 1 h of its obtaining, which apparently contributed to the successful outcome.

Improving the efficiency of the OTO-IVM method by increasing the proportion of mature oocytes is a key objective. An important achievement can be considered the two-phase IVM (CAPA-IVM) method, which allows avoiding the desynchronization of nuclear and cytoplasmic maturation, thereby increasing the proportion of competent oocytes. A case of live birth is described from oocytes obtained via transvaginal ovarian puncture, with subsequent maturation using the CAPA-IVM method (23). However, it should be noted that, at present, the vast majority of reports of live births following IVM are associated with the use of the standard single-phase method. Unfortunately, we are not aware of any registered commercial culture media for two-phase IVM, which is a necessary condition for its clinical application.

As contrasted with the first cases of childbirth after OTO-IVM, we were able to transfer an embryo at the blastocyst stage for the first time. Of course, after OTO-IVM, obtaining embryos with high development potential is an extreme challenge for the embryologist. The survival rate of embryos with reduced developmental potential is higher *in vivo*, which may explain the transfer at the cleavage stage in the cases described earlier.

The fact of obtaining embryos serves as strong motivation for cure from oncological disease. Possibly, in many cases described in literature regarding cryopreservation of embryos at the cleavage stage, authors had concerns that the embryo might fail to develop into a blastocyst, resulting in loss of biological material necessary for future restoration of fertility. Thus, they considered it a better strategy to preserve the embryo at an earlier developmental stage. We considered it important to have viable embryos with a high implantation potential, which is the blastocyst stage, in order to minimize the risk of a futile transfer. It is our belief that cultivation to blastocyst stage is significantly more effective, both in terms of increasing the likelihood of live birth per transfer and in terms of the need for preimplantation genetic testing in some clinical cases. Cultivation with the use of time lapse allowed us to evaluate the morphokinetic parameters of embryo development and assess their potential for implantation as quite high (24) Preimplantation testing of embryos for monogenic diseases seems to be particularly important for cancer patients to prevent the transmission of genetically determined forms of cancer to offspring, such as those caused by mutations in the BRCA1 and 2 genes. In such cases, the optimal strategy would be cultivation to blastocyst stage followed by trophectoderm cell biopsy and their genetic testing. Performing PGT-A is not indicated for young female patients, but in some cases, surrogacy is used, and given the complexity of all aspects of preparation for this procedure, patients are often interested in transferring an euploid embryo after PGT-A to increase the chances of getting pregnancy. The feasibility and importance of obtaining euploid embryos in OTO-IVM programs have been demonstrated. Although these embryos have not yet resulted in a live birth, their availability serves as a significant motivating factor for the patient (19). On the other hand, even if the embryo is transferred to the patient herself, maximizing the chances of implantation may be extremely important, since hormonal support in patients with a history of hormone-sensitive tumors may become an additional risk factor for recurrence. In addition, information about higher chances of implantation of the transferred embryo can have a positive effect on the female patient’s psychological state.

The clear limitation of our report is that we present here a single successful case. We recognize that each case is unique and the method remains experimental. Standardization of all stages, accumulation of data, and the development of guidelines are required. It is extremely important to inform patients before the procedure so that they understand the chances and limitations of the method.

By presenting our work, we insist that cultivating an embryo to the blastocyst stage by fertilizing an oocyte obtained by OTO-IVM from an ovary affected by a malignant or borderline tumor is possible and has the potential for a successful pregnancy and the birth of a healthy child. It is our belief that further research is required in the line of improving OTO-IVM protocols to increase the effectiveness of the method.

## Data Availability

The datasets presented in this article are not readily available because. Requests to access the datasets should be directed to jul_taty@mail.ru.

## Supplementary material

Supplementary Figure 1 Embryo (4 AA) after thawing before transfer into the uterine cavity. The image was taken under an Olympus IX73 inverted microscope (Olympus Corporate, Japan) at 400x magnification.
