# Supplementary figures and images for: Clinical case: successful birth as a result of the transfer of an embryo at the blastocyst stage from an oocyte obtained from a tumoral ovary using the OTO-IVM method
Source: Front Med (Lausanne). 2026 Jul 17;13:1857432. doi: 10.3389/fmed.2026.1857432 (PMC13423897; doi:10.3389/fmed.2026.1857432)

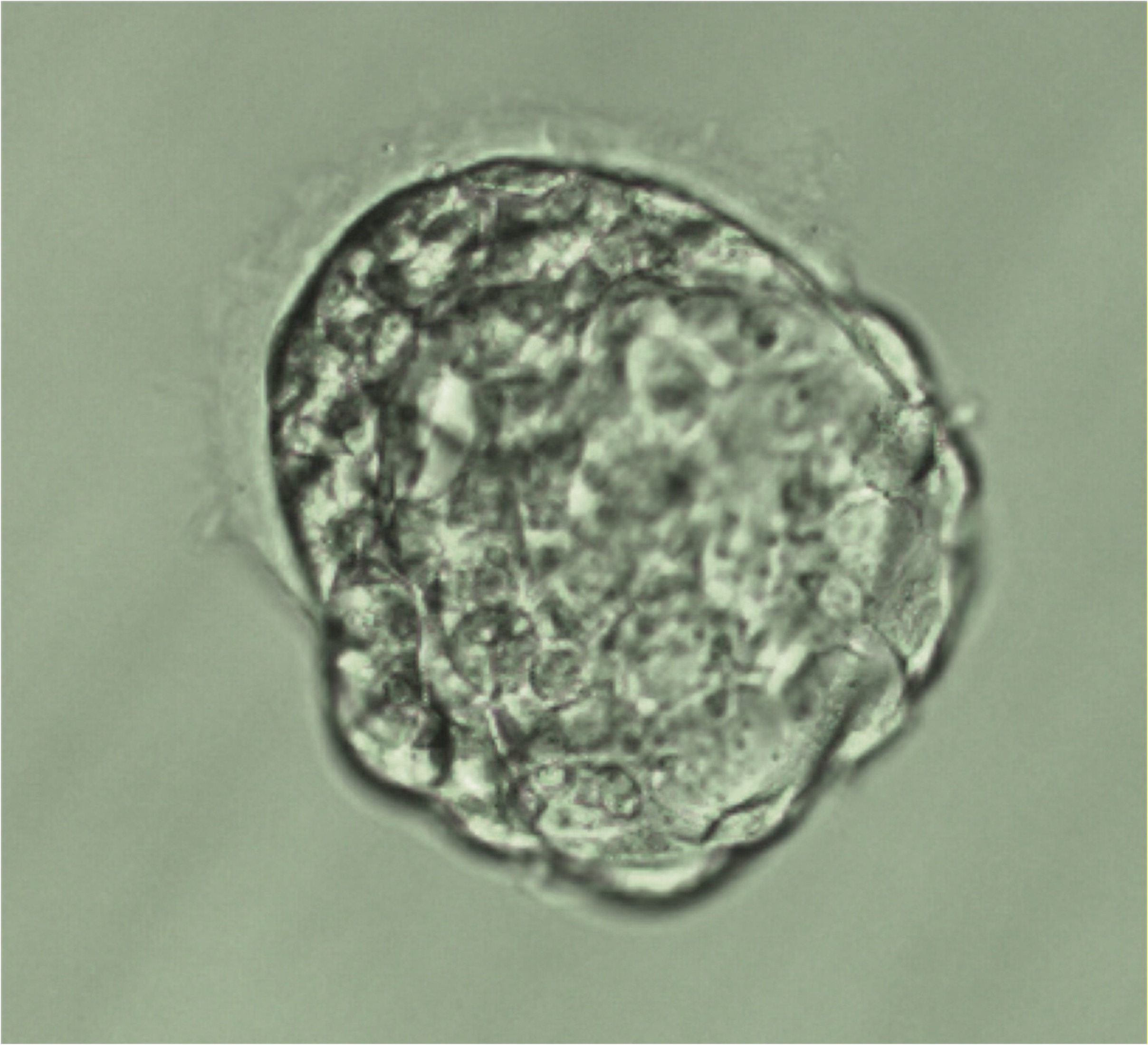

Supplement: Supplementary Figure 1 — Embryo (4 AA) after thawing before transfer into the uterine cavity. The image was taken under an Olympus IX73 inverted microscope (Olympus Corporate, Japan) at 400x magnification. [file Image_1.jpeg]
